# Robotic-assisted surgery in extremely obese patients: a multidisciplinary approach for a patient with a BMI of 101.7 kg/m^2^

**DOI:** 10.1007/s00404-025-08158-5

**Published:** 2025-08-26

**Authors:** Roland Csorba, Sàed Almasarweh, Zeynep Atas Elfrink, Paul Buderath, Rainer Kimmig, Martin W. Britten

**Affiliations:** 1 Department of Obstetrics and Gynaecology, University Clinic Essen, University of Duisburg-Essen, Hufelandstr. 55, 45147 Essen, Germany; 2 Department of Anaesthesiology and Intensive Medicine, University Clinic Essen, University of Duisburg-Essen, Essen, Germany

**Keywords:** Endometrial cancer, Morbid obesity, Robotic surgery, Cancer-field surgery, Multidisciplinary management, Anaesthesiology

## Abstract

**Background:**

The prevalence of obesity has risen significantly, affecting over 19% of the German population. Obesity is frequently associated with endometrial cancer, presenting considerable challenges in pre-, intra- and postoperative management. Challenges with intubation, patient positioning, respiratory and cardiac complications as well as wound dehiscence are commonly encountered in this patient population.

**Methods and results:**

For patients with uterine cancer, surgical intervention is essential for staging, symptom control, and potential cure. Minimally invasive approaches, particularly robotic-assisted surgery, have expanded the possibilities for treating morbidly obese patients. Robotic systems facilitate navigation around anatomical barriers and reduce surgeon fatigue. However, despite the technological advancements, morbidly obese patients often face increased perioperative risks and prolonged postoperative recovery. Laparoscopic procedures in steep Trendelenburg position for morbidly obese patients pose unique challenges, particularly in anesthesiological management. These challenges necessitate individualized ventilatory and hemodynamic support to ensure patient safety.

This case highlights a multidisciplinary approach to managing a patient with extreme obesity (BMI 101.7 kg/m^2^) undergoing roboticassisted surgery for uterine cancer. It underscores the importance of comprehensive preoperative planning, intra-operative considerations, and post-operative care in minimizing complications and optimizing outcomes.

**Conclusion:**

Our case exemplifies our experience from similar cases and demonstrates that robotic-assisted surgery for endometrial cancer in obese patients can represent a safe and feasible option, characterized by a low complication rate, minimal blood loss, and a short hospital stay.

## What does this study adds to the clinical work

**Table:** 

Obesity is a significant risk factor for endometrial cancer, and its increasing prevalence underscores the importance of developing effective surgical techniques to manage these patients.	
Laparoscopic procedures in the steep Trendelenburg position for morbidly obese patients pose unique challenges, particularly in anesthesiological management. These challenges necessitate individualized ventilatory and hemodynamic support to ensure patient safety.	
Robotic-assisted surgery for endometrial cancer in obese patients has proven to be safe and feasible option characterized by a low complication rate, minimal blood loss, and a short hospital stay.	

## Introduction

Obesity affects approximately 19% of the German population [1]. A body mass index (BMI) of 40 or higher classifies a patient as obesity class III, signifying a high risk of obesity-related diseases and a significant impact on quality of life. Obesity is frequently associated with endometrial cancer and presenting challenges in surgical management [2]. The intersection of these conditions creates a complex clinical scenario requiring careful consideration. Obesity increases the risk of complications such as wound dehiscence, respiratory and cardiac issues, and challenging intubations. In patients with uterine cancer, surgery remains essential for both staging and treatment. The introduction of the DaVinci™ robotic surgical system has expanded the options for minimally invasive procedures, offering potential benefits for high-risk patients.

While surgery is the cornerstone for managing uterine cancer, morbidly obese patients experience increased perioperative complications and prolonged post-operative recovery [3]. In this context, a minimally invasive approach, enhanced by robotic assistance, can provide a viable alternative. We report the case of a patient with a BMI of 101.7 kg/m^2^ who underwent a robotically assisted total laparoscopic hysterectomy performed as peritoneal mesometrial resection (PMMR) and bilateral salpingo-oophorectomy with pelvic-targeted compartmental lymphadenectomy (TCL) using the DaVinci™ robotic system.

This case represents the largest patient, weighing 287 kg, to undergo robotic cancer-field surgery. We emphasize the multidisciplinary management approach, involving gynecologic oncology and anesthesiology to successfully address the unique challenges posed by this complex case. To our knowledge, this is the largest patient reported in the literature to undergo a robotic procedure of this nature.

## Case report

A 57-year-old nulligravid Caucasian female with a BMI of 101.7 kg/m^2^ (height 168 cm, weight 287 kg) presented with postmenopausal vaginal bleeding persisting for 4 months. Her medical history was notable for arterial hypertension, recurrent abscesses, hypothyroidism following thyroidectomy, psychogenic eating disorder, anxiety, and borderline personality disorder. Her surgical history included laparoscopic ovarian cyst removal (1998), dilation and curettage (2000), and thyroidectomy (2005).

The patient was not sexually active, and her Eastern Cooperative Oncology Group (ECOG) performance status was 1. On physical examination, she was 168 cm tall and weighed 287 kg, with circumferences of 130 cm and 118 cm for the right and left thighs, respectively. Bilateral pitting edema grade 2 + was observed in her thighs. Otherwise, her general and pelvic examinations were unremarkable.

Her initial laboratory evaluation revealed hemoglobin of 12.4 g/dl, mean corpuscular volume (MCV) of 89.8 fl, and CA 12–5 levels of 22 U/ml. Her Pap smear showed no abnormalities.

A pre-operative CT of the thorax and abdomen demonstrated an asymmetrical uterine form and enlarged retroperitoneal lymph nodes. The lung fields were clear with no evidence of metastatic disease, and the heart size was within normal limits. A CT of the skull showed no signs of metastasis.

Given her immobility and significant comorbidities, the patient displayed signs consistent with congestive heart failure, leading to a high perioperative risk. To evaluate cardiac function, transthoracic echocardiography was performed. The findings included pulmonary hypertension with a systolic pulmonary artery pressure of 38 mmHg (plus central venous pressure), moderate tricuspid valve insufficiency, and a preserved left ventricular ejection fraction of 50%. Mild mitral valve insufficiency was also noted and the aortic valve appeared normal.

Based on these findings, the patient was classified as ASA IV according to the American Society of Anesthesiologists (ASA) Physical Status Classification System, indicating a severe systemic disease posing a constant threat to life.

To perform the dilation and curettage (D&C), the patient’s legs were placed on two stands, as they could not be positioned in stirrups for the lithotomy position due to their size. Post-operatively, the patient experienced a hypertensive crisis with a blood pressure of 240/100 mmHg, which was successfully managed with standard drug therapy. Histopathological analysis of the D&C specimen revealed endometrioid adenocarcinoma, FIGO grade 1.

Subsequently, the patient underwent a robotically assisted total laparoscopic hysterectomy performed as peritoneal mesometrial resection (PMMR) and bilateral salpingo-oophorectomy with pelvic-targeted compartmental lymphadenectomy (TCL).

**Fig. 1:**
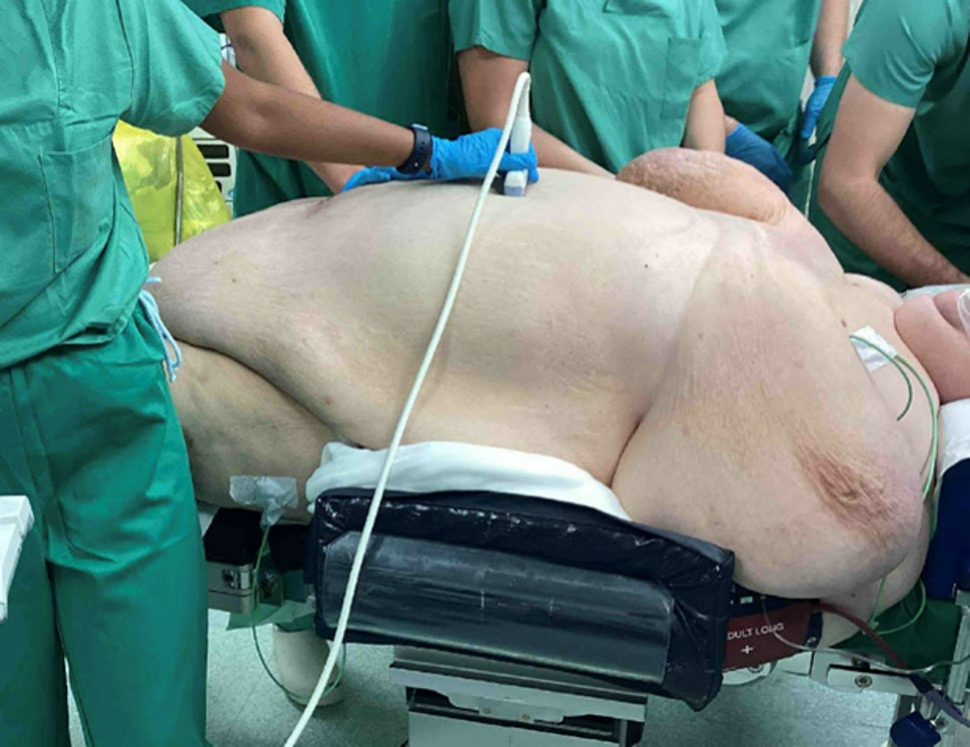
A specialized surgical bed was utilized to accommodate the patient’s unique anatomical considerations. The patient was positioned supine

**Fig. 2:**
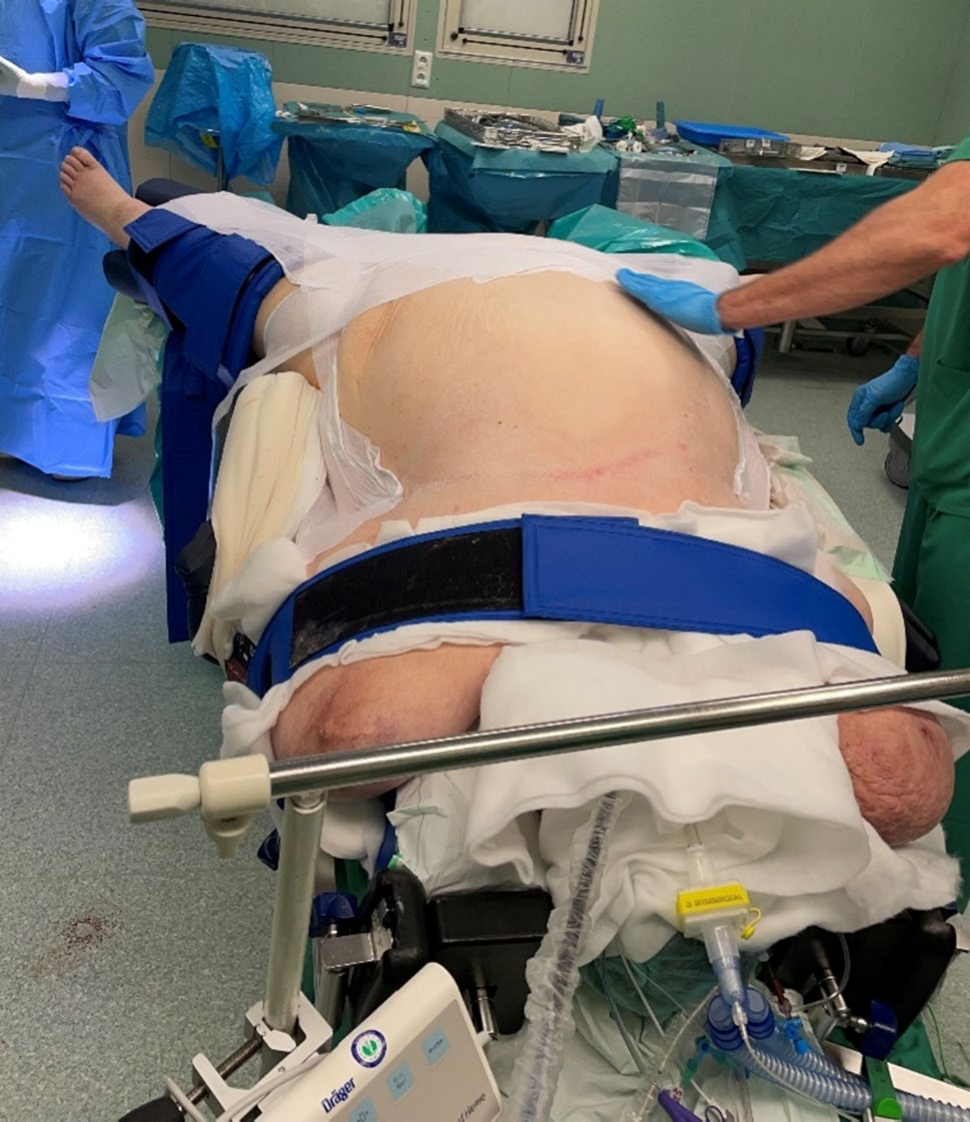
The tilt of the table was positioned at 30-degree and the final position was checked before docking the robot

The patients’ position on the operating table was standardized. Patients were positioned on a bean bag to prevent them from slipping during the intervention. A specialized surgical bed was utilized to accommodate the patient’s unique anatomical considerations. The surgical bed was padded with egg crate foam along its surface and edges to ensure pressure relief and minimize the risk of pressure-related injuries. Multiple bed extenders were attached to accommodate the width of the patient’s torso and legs. Due to the size of her legs, the patient could not be placed in the lithotomy position and was instead positioned supine. The arms were fixed on the side of the patients and shoulder supports were also placed to ensure additional protection. Circumferential padding and taping were applied around her lower legs and chest for additional stabilization and support. Arm sleds were repurposed to support her lower legs, positioned adjacent to the bed, ensuring optimal access for the surgical procedure (Fig. 1). The tilt of the table was positioned at 30-degree and the final position was checked before docking the robot (Fig. 2).

Indocyanine green (ICG) was applied to the corpus myometrium. An IOWA trumpet as used for pudendal anesthesia in obstetrics was inserted into the corpus transcervically, and a total of 2 mL ICG-solution (1.66 mg/mL) was injected into four different sites, fundus right/left and midcorporal right/left, 0.5 mL/each. To avoid perforation and thus contamination of the situs, injection was performed under laparoscopic visual control following coagulation of the fallopian tubes. Cervical channel was closed by a suture and additionally by an alcohol sponge to prevent cell spilling.

A HOHL uterine manipulator was successfully placed in the vagina to distend the vaginal fornices and assist in performing the colpotomy. Long robotic trocars (120 mm length × 8 mm) were used to access the abdominal cavity and were configured in a five-trocar arrangement. The patient and bed were positioned at a 30° Trendelenburg angle, and the robotic system was docked at an oblique angle from the patient’s right side.

Monopolar scissors, a PK coagulator, and Maryland graspers were employed via the robotic arms, while a 30° scope provided optimal visualization. Insufflation pressure was maintained between 12 and 15 mmHg throughout the procedure, with a reduction in pressure at the end to facilitate ventilation by the anesthesia team.

The combination of the steep Trendelenburg position and pneumoperitoneum-induced hypercapnia has the potential to exacerbate pulmonary artery pressure and increase the risk of heart failure. To mitigate these risks, advanced hemodynamic monitoring was implemented, including continuous transesophageal echocardiography and the placement of arterial, central venous, and pulmonary artery catheters.

**Fig. 3:**
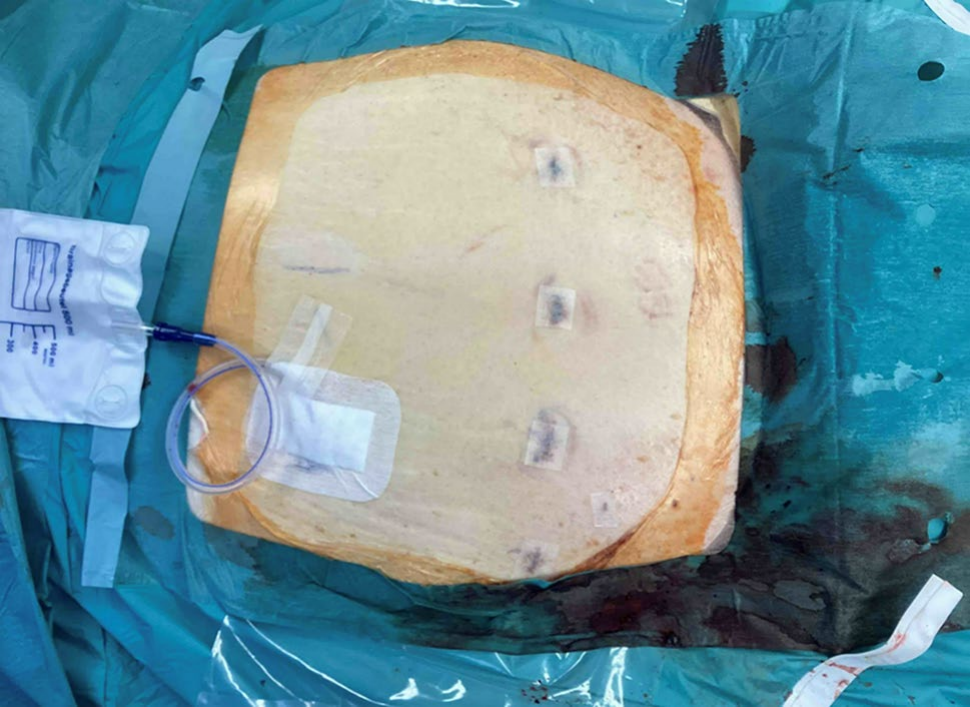
Postoperativ situs with intra-abdominal drain after removing the trocars

Using this setup, a robotically assisted total laparoscopic hysterectomy and bilateral salpingo-oophorectomy with indocyanine green-near-infrared fluorescence guided, targeted sentinel lymphadenectomy could be performed. The uterus, fallopian tubes, ovaries, and lymph nodes were removed vaginally using an 800 mL Endobag. The vaginal cuff was closed robotically with a continuous V-Loc suture. An intra-abdominal drain was placed in the pelvis. Figure 3 shows the situation after skin closure.

Positioning and anesthesia time totalled 131 min, while the surgical procedure lasted 264 min. The overall operating room time was 437 min. The estimated blood loss during the procedure was 150 ml. Pre-operatively, the patient received 3 g of ampicillin/sulbactam. Due to the high risk of pulmonary complications, the patient was admitted to the intensive care unit post-operatively. She was extubated and initially supported by non-invasive ventilation for several hours before being transferred to the general ward on the second post-operative day. Her post-operative course was uncomplicated. The patient was discharged on the twenty-second post-operative day, primarily due to non-gynecological chronic conditions, including mild vision loss from hypertensive retinopathy, as well as the need to address issues related to her immobility and plan post-discharge care. The patient declined our recommendation to present her case for bariatric surgery.

Final pathology revealed a tumor in the uterus measuring 5.4 × 5.2 × 2.6 cm, diagnosed as FIGO grade 1 MSS endometrioid adenocarcinoma, p53 wild type, with 1 cm of myometrial invasion. No lymphovascular space invasion (LVSI) was identified. The final TNM classification was pT3a pN0 (0/1) L0 V0 Pn0 R0 G2.

Further treatment recommendations included radiotherapy and adjuvant chemotherapy with carboplatin and paclitaxel administered intravenously every 3 weeks. Post-operatively, the case was discussed in the interdisciplinary conference and the patient was referred to radiation oncology to discuss adjuvant treatment options. During the consultation, various approaches were reviewed, including whole pelvic radiation, vaginal cuff brachytherapy (VBT), and a combination of both. After a thorough discussion of the risks and benefits of all options, the patient expressed significant concerns about the potential toxicity of the recommended therapy. Having undergone cancer-field surgery she decided to omit post-operative treatment.

## Discussion

Obesity is a prevalent chronic disease, and morbid obesity is an increasingly alarming global problem. In Germany, 53.5% of the population (46.6% of women and 60.5% of men) is classified as overweight, including those with obesity. Obesity affects 19.0% of adults [1]. The management of extreme obesity—defined as a body mass index (BMI) ≥ 50 kg/m^2^ or obesity classes IV, V, and VI—remains a significant challenge. Individuals with extreme obesity face an elevated risk of surgical complications, increased mortality, and multiple comorbidities.

Endometrial cancer (EC) is a malignancy of the inner epithelial lining of the uterus, with a rising global incidence and associated mortality [4]. In Germany, EC is the most common gynecological malignancy. In 2020, there were 417,336 new cases of EC worldwide, making it the sixth most commonly occurring cancer among females [5]. The majority of cases occur between the ages of 65 and 75 years [6]. This type of cancer comprises a range of distinct histological subtypes and molecular phenotypes. Historically, EC has been classified into two categories: Type I, which is associated with unopposed estrogen stimulation, features low-grade cells, is more common, and has a favorable prognosis; and Type II, which is not estrogen-driven, features high-grade cells, is less common, and has a poorer prognosis. Type I EC primarily includes grade I or grade II endometrioid adenocarcinomas, while Type II encompasses grade III endometrioid adenocarcinomas, serous carcinoma, clear cell carcinoma, undifferentiated carcinoma, and carcinosarcomas [7].

Several factors increase the risk of developing EC, including advanced age, certain ethnicities, higher BMI, endogenous or exogenous estrogen exposure, tamoxifen use, early menarche, late menopause, lower parity, metabolic syndrome, family history, and genetic predisposition. Conversely, factors such as maintaining a normal BMI, having higher parity, and using oral contraception are associated with a lower risk of EC [7].

To our knowledge, the case presented here involves the largest patient to undergo surgery for endometrial cancer using the DaVinci™ robotic system. This case highlights the feasibility and advantages of robotically assisted surgery in patients with morbid obesity even for complex procedures such as the oncological cancer-field surgery presented here. As cancer-field surgery by PMMR and TCL aims at reaching optimal locoregional control without the need for adjuvant radiotherapy, it may help to minimize treatment-related risks for this extremely vulnerable collective by avoiding both laparotomy as well as irradiation. Conventional laparoscopy is often impractical for such complex procedures while open surgery via laparotomy carries a significantly increased perioperative risk in this patient population [8]. A robotic-assisted approach provides a magnified, three-dimensional view of the surgical site, along with improved dexterity and range of motion, allowing for greater precision during such complex procedures as the depicted PMMR and TCL. The position of the surgeon at the console poses less physical strain and therefore improves the capability to perform enduring and complex procedures. Especially in patients with difficult surgical conditions, robotic arms can navigate to hard-to-reach areas with greater flexibility and less tissue disruption. Our experience from similar cases shows that after placement of the patient in steep Trendelenburg position, the surgical view from the console is only slightly different from the one in a non-obese patient, enabling the surgeon to offer a surgical therapy, which most likely cannot be offered with conventional laparoscopy.

Although the robotic-assisted laparoscopic approach is advantageous for morbidly obese patients, it presents significant anesthesiological challenges. Obesity reduces functional residual lung capacity and chest wall compliance, increasing airway resistance and the risk of atelectasis. These issues are exacerbated by the steep Trendelenburg position [9]. Ventilating a morbidly obese patient in a 30° Trendelenburg position becomes even more challenging with the addition of capnoperitoneum. To mitigate respiratory insufficiency, the application of adequate positive end-expiratory pressure (PEEP) is crucial [10].

In this case, optimal PEEP was determined through titration based on dynamic lung compliance. The patient was ventilated using pressure-controlled, low-tidal-volume ventilation of 7 ml/kg ideal body weight, with a respiratory rate of 20 breaths per minute and an inspiratory-to-expiratory ratio of 1:1. The respiratory rate was further adjusted based on end-tidal capnometry. Following an initial recruitment maneuver, optimal compliance was achieved with a PEEP of 24 mbar and a peak airway pressure of 42 mbar. With a respiratory rate of 28 breaths per minute, a minute volume of 10.9 L was maintained.

While high PEEP values in morbidly obese patients improve oxygenation, they do not necessarily lead to pulmonary barotrauma. Barotrauma depends on excessive transpulmonary pressure, which is calculated as the difference between intra-alveolar and intrapleural pressure. In this case, the elevated intrapleural pressure caused by the patient’s body weight in the steep Trendelenburg position, combined with increased intra-abdominal pressure during laparoscopy, allowed for higher intra-alveolar pressures to be well-tolerated. These pressures minimized atelectasis formation and potentially improved post-operative pulmonary function [11].

This approach—using a higher PEEP—reduces driving pressure during ventilation, a factor strongly associated with improved post-operative pulmonary outcomes [12]. In addition, low-tidal-volume ventilation of 7 ml/kg ideal body weight was shown to enhance post-operative outcomes, though it may complicate the maintenance of normocapnia [13]. In this case, arterial hypercarbia up to 67 mmHg was tolerated, as arterial pH remained above 7.2. Oxygenation was well-maintained, with an FiO₂ of 0.5 yielding arterial pO₂ values above 80 mmHg.

Apart from the challenges associated with ventilation, hemodynamic changes during the steep Trendelenburg position are also significant, including increases in arterial and central venous pressures, cardiac output, systemic vascular resistance, and pulmonary artery pressure^8^. These changes may be further aggravated by high PEEP values and hypercarbia. With a pulmonary artery catheter in place, the mean pulmonary artery pressure in the supine position was initially measured at 45 mmHg, which increased to 69 mmHg during surgery. Inhalation of 20 µg Iloprost as a pulmonary vasodilator did not result in a significant decrease in pulmonary artery pressure. However, transesophageal echocardiography revealed that cardiac function remained compensated without signs of right heart failure, allowing surgery to proceed after a slight decrease in capnoperitoneal pressure. Arterial pressure was maintained through continuous infusion of noradrenaline. Given that we did not expect significant blood loss during surgery, infusion of balanced crystalloids was limited to a total of 500 mL.

Although the patient’s respiratory and hemodynamic status after surgery remained uneventful, we anticipated an increased risk of post-operative pulmonary complications. Post-operative pulmonary complications have been reported in 19% of patients following robotic-assisted surgery, and morbid obesity is known to further elevate this risk [14]. Consequently, the patient was transferred to our intensive care unit, where she was extubated and initially supported by non-invasive ventilation to optimize and maintain respiratory function. She was subsequently discharged from the ICU on the second post-operative day without incident.

In summary, we report a case of multidisciplinary management for an extremely obese patient undergoing robotically assisted oncological surgery. This case is unique because it demonstrates how robotic surgery can address the challenges posed by extreme obesity. The complexities of surgical and anesthesiological management necessitate a multidisciplinary approach, often involving specialists in gynecologic oncology and anesthesiology. Future studies are needed to address the specific needs of patients with extreme obesity, as their outcomes are likely to differ from those of patients with a lower BMI.

## Conclusion

Obesity is a significant risk factor for endometrial cancer, and its prevalence continues to rise. As such, the development of surgical techniques to manage these patients is crucial. Minimally invasive surgery, particularly with robotic assistance, has expanded the possibility of performing surgeries on morbidly obese women. It enables surgeons to navigate around anatomical barriers and reduces surgeon fatigue. While it presents complex challenges for anesthesiologists, surgical nurses, and surgeons, these challenges can be managed through individualized and interdisciplinary approaches. With regard to anesthesia, optimized ventilation with adequate PEEP is critical for preventing hypoxemia and excessive hypercapnia. In addition, individualized management of hemodynamic changes, particularly in patients with known cardiac dysfunction, is essential. Given the rising rates of obesity and the high prevalence of uterine cancer, there is a growing need for safe and effective therapeutic options for this population. Our case depicts robotic-assisted surgery for endometrial cancer in class VI obese patients as a safe and feasible option, offering low complication rates and comparable oncologic outcomes. While the prevalence and severity of obesity are rising, robotic-assisted surgery may enable surgeons to offer such complex procedures as depicted as a therapeutic choice for the management of early stage endometrial cancer even in extremely obese patients.

## Data Availability

No datasets were generated or analyzed during the current study.
